# 16S rRNA gene profiling of bacterial communities mediating production of tsetse attractive phenols in mammalian urine

**DOI:** 10.4102/ojvr.v86i1.1724

**Published:** 2019-07-17

**Authors:** Harry A. Musonye, Ezekiel M. Njeru, Ahmed Hassanali, Lydia M. Langata, Dominic Mijele, Titus Kaitho, Edward King’ori, James Nonoh

**Affiliations:** 1 Department of Biochemistry, Microbiology and Biotechnology, Kenyatta University, Nairobi, Kenya; 2 Department of Chemistry, Kenyatta University, Nairobi, Kenya; 3 Department of Veterinary Services, Kenya Wildlife Service, Nairobi, Kenya

**Keywords:** trypanosomiasis, tsetse, phenols, bacteria, mammalian urine

## Abstract

Several types of odours are involved in the location of host animals by tsetse (Diptera: Glossinidae), a vector of animal African trypanosomiasis. Host animals’ ageing urine has been shown to be the source of a phenolic blend attractive to the tsetse. Nevertheless, limited research has been performed on the microbial communities’ role in the production of phenols. This study aimed at profiling bacterial communities mediating the production of tsetse attractive phenols in mammalian urine. Urine samples were collected from African buffalo (*Syncerus caffer*), cattle (*Bos taurus*) and eland (*Taurotragus oryx*) at Kongoni Game Valley Ranch and Kenyatta University in Kenya. Urine samples, of each animal species, were pooled and left open to age in ambient conditions. Bacteriological and phenols analyses were then carried out, at 4 days ageing intervals, for 24 days. Phenols analysis revealed nine volatile phenols: 4-cresol, ortho-cresol, 3-cresol, phenol, 3-ethylphenol, 3-propylphenol, 2-methyloxyphenol, 4-ethylphenol and 4-propylphenol. Eight out of 19 bacterial isolates from the ageing urine revealed the potential to mediate production of phenols. 16S rRNA gene characterisation of the isolates closely resembled *Enterococcus faecalis* KUB3006, *Psychrobacter alimentarius* PAMC 27887, *Streptococcus agalactiae* 2603V, *Morganella morganii* sub.sp. *morganii* KT, *Micrococcus luteus* NCTC2665, *Planococcus massiliensis* strain ES2, *Ochrobactrum pituitosum* AA2 and *Enterococcus faecalis* OGIRF. This study established that some of the phenols emitted from mammalian urine, which influence the tsetse‘s host-seeking behaviour, are well characterised by certain bacteria. These results may allow the development of biotechnological models in vector control that combines the use of these bacteria in the controlled release of semiochemicals.

## Introduction

Animal African trypanosomiasis (AAT) has for years continued to suppress livestock health and productivity in sub-Saharan Africa. It is an endemic parasitic disease of domestic livestock with negative impact on people’s income on the African continent. The disease covers about 11 million square kilometers of sub-Saharan Africa occurring in 37 countries (Cecchi et al. 2009). Cattle, sheep, camels, goats and pigs are the main economically important domestic species in which the infection is most commonly diagnosed (Auty et al. 2015). The direct and indirect cost caused by this disease is estimated in billions of dollars (Chitanga et al. 2011).

Four biological factors have been described as variables that manipulate epidemiology of AAT in tsetse infested areas of Africa. These factors are parasite, vector, livestock and reservoir hosts (Van den Bossche et al. 2010). *Trypanosoma* species are the causative parasites for AAT. The species include *Trypanosoma vivax, Trypanosomabrucei brucei* and *Trypanosoma congolense* (Lai et al. 2008). These parasites cause moderately mild infections in wild animals as compared to domestic animals where they cause cruel, and often fatal disease. Currently, there is little evidence of successful vaccine development for the trypanosomes as the parasite apparently has the capacity to evade mammalian immune defences (Esterhuizen et al. 2011; Cnops, Magez & De Trez 2015; Scolari et al. 2016). Control of AAT as a measure to improve the production capacities of rural-based communities in regions infested by tsetse has been addressed by other methods. The methods primarily include use of insecticides, trypanocidal drugs, trapping vectors, pesticide treatments and sterile male release strategies (Hargrove et al. 2012; Holmes 2013; Shaw et al. 2015). Nevertheless, these methods have their own weaknesses. For instance, use of trypanocidal drugs has been limited by efficacy factors because of multiple drug resistance (Moti et al. 2015). Another key concern is fake and substandard drugs as well as drug safety, in terms of residues in food-producing animals (Baker et al. 2013). Spraying of insecticides in the environment is expensive and toxic and drives the evolution of resistance in target vectors (Scolari et al. 2016; Touré, Ramirez & Sommerfeld 2015).

Tsetse fly species (Diptera: Glossinidae) belonging to the genus *Glossina* are the main vectors of trypanosomes. The species are subgrouped into morsitans, palpalis and fusca that inhabit savannah, riverine environments and forests in that order (Rayaisse et al. 2011). Of the three groups of *Glossina* spp., the savannah and riverine groups inhabit areas suitable for grazing and watering. They live among livestock and wildlife where they feed on bloodstream meal from infected hosts and pick trypanosomes, the AAT disease causing parasites. The tsetse flies’ ability to detect and locate suitable hosts from which to feed on is critical for their survival and reproduction (Lord et al. 2017). They achieve this through short-range visual and long-range odour sensation (Omolo et al. 2009).

Different odour sources that include, but are not limited to, urine, faeces, skin surface, manure and the breath of cattle have been shown to enhance host location by the tsetse vector. Among these, mammalian urine and the breath of cattle have been researched. In their studies, Alkhaldy, Edwards and Combet (2018), Spiehs et al. (2018) and Tangtrakulwanich et al. (2015) identified phenols in mammalian urine. According to Hassanali et al. (1986) and Baldacchino et al. (2014), mammalian urine is composed of phenolic compounds that are odorous and highly attractive to tsetse. Among the several phenols present in the urine of mammals, for instance, cattle or buffaloes, 4-cresol and 3-n-propylphenol are the most attractive to *Glossina* spp. (Madubunyi et al. 1996; Owaga et al. 1988; Saini 1990). However, for riverine tsetse, these phenols are not very attractive (Hall et al. 1990). Tsetse attractant phenols in mammalian host urine are believed to form slowly from the pro-attractants (Mihok & Lange 2012). These attractants have been identified as a combination of sulphates, glucuronates and other unabsorbed precursors in urine. Studies have suggested the involvement of microbial communities in the breakdown of these conjugates into tsetse attractive phenols (Omolo et al. 2009). The attraction of tsetse species to livestock and wildlife is therefore attributable to phenolic compounds in urine. It is essential to, therefore, profile the bacterial communities associated with the production of tsetse attractive phenols in mammalian urine to help in the generation of intervention tools in AAT control and elimination.

## Materials and methods

### Study area

Sample collection was carried out at the Kongoni Game Valley Ranch (0.7754° S, 36.3715° E) and the Kenyatta University Cattle Farm (1.1767° S, 36.9365° E) in Kenya. The Kongoni Game Valley Ranch is situated in an agro-pastoralist semi-arid ecosystem and consists of mostly Acacia woodland with thickets along Lake Naivasha. The Kenyatta University Cattle Farm is characterised by a semi-intensive farming system.

### Urine sample sources

Disease-free male and female African buffaloes (*Syncerus caffer*), domestic cattle (*Bos taurus*) and eland (*Taurotragus oryx)* were used in this study. The choice of these species was influenced by the reported presence of tsetse attractive phenols in cattle and buffalo urine (Hassanali et al. 1986; Madubunyi et al. 1996). Eland have been shown to be resistant to trypanosomiasis (Pappas 2002). However, it has not been known if the animal’s urine has the potential to attract tsetse. It was therefore thought to be of interest to determine if there are tsetse attractant cues and their bio-agents in the urine.

### Urine sample collection

Fresh buffalo and eland urine samples were collected in collaboration with the Kenya Wildlife Service Veterinary Department. Collection was opportunistic, as the selected wildlife urinated naturally, during de-snaring and translocation activities. Fresh cattle urine was also collected while the animals were urinating naturally. Sterile 50 mL universal bottles were used in collecting the urine samples. Samples were collected over a period of 2 weeks. The samples were immediately placed in a cool box at 4 °C and transferred to the Kenyatta University Microbiology Research Laboratory for bacteriological analysis and the preparation of phenols analysis.

### Sample preparation

Once in the laboratory, the 50 mL bottle urine portions collected for the 2 weeks, for each animal species, were aseptically pooled together into sterile transparent plastic bottles measuring 1000 mL. The total pooled samples for each species measured about 800 mL. These pooled samples, for each species, were then divided into two, each measuring 400 mL. One was preserved at -20 °C to defer any chemical transformation, and the other was left open and allowed to age in ambient conditions, throughout the study period (24 days), in the laboratory.

### Evaluation of phenols generation in mammalian urine samples at ageing intervals

#### Extraction and clean-up phase of mammalian ageing urine

The liquid–liquid extraction (LLE) method was used in the extraction. Briefly, the hydrophobic organic phase of pooled urine samples for each animal was extracted using analytical grade dichloromethane (DCM) (Loba Chemie, Mumbai India), three times (30 mL, 30 mL and 30 mL), at intervals of 4 days, for 24 days. During the extraction, 50 mL of urine was put in a separating funnel, mounted on a stand, with the stopcock at the bottom closed; 30 mL of DCM was then added to the urine sample, in the separating funnel, to form two layers. The contents in the separating funnel were then shaken thoroughly, four times, with thumb held firmly on the stopper. During the shaking, the stopcock was opened periodically to vent the vapour pressure build up. The separating funnel was then mounted back on the stand. The stopper was taken off, the stopcock opened and the lower organic layer was drained into a beaker. These steps were repeated with two more 30 mL DCM portions.

The bottom organic layers for each fermenting interval, of each study animal, were pooled together and put in conical flasks. The top hydrophilic aqueous layers were also kept separate until extraction was complete and the product isolated. Further purification of the organic layers was realised using anhydrous sodium sulphate (Na_2_SO_4_), a drying agent. The agent was added to the organic phase in a conical flask using a spatula and swirled gently. The sulphate was added until it was seen to form floating solids from an initial clump like appearance. The mixture was left to stand for about 5 minutes to ensure complete absorption of water. Complete absorption of water was determined to have been achieved after the extracted contents turned into a clear liquid, from a cloudy like solution. The dried extracted organic layer was decanted and transferred to a beaker. The layer was then subjected to a rotary pump at 40 °C to concentrate the sample. All the DCM was evaporated until organic compounds, in forms of wax-like substances, were formed at the walls of the rotary flask. Thereafter, 2 mL of DCM was added into the rotary flask and swirled round to ensure collection of the wax-like substance from the walls. The substances for each sample were passed through a 0.22 *µ*m Millipore filter and the contents transferred to bijou bottles, wrapped in aluminium foils, and kept at –20 °C for subsequent analysis.

#### Gas chromatography-mass spectrometry analysis

For the gas chromatography-mass spectrometry (GC-MS) analysis, a stock solution was prepared. In this preparation, 1 mg of a sample was separately weighed and dissolved in 1 mL DCM to make a stock solution, 1 mg/mL, from which an experimental sample with a final concentration of 100 ng/*µ*L was prepared. In addition, 1 mL of internal standard, a control solution, was aliquoted and analysed.

The samples were analysed by GC-MS on an ISQ 7000 (Thermo Fisher Scientific, Massachusetts, United States [US]), using the following operating conditions: (1) Transfer line temperature of 280 °C, Inlet temperature of 270 °C and column oven temperature programmed from 35 °C to 280 °C with the initial temperature maintained for 5 min, then 10 °C/min to 280 °C for 10.5 min and the final one at 50 °C/min to 285 °C for 29.9 min. (2) A HP-5 MS low-bleed capillary column fitted GC (30 m × 0.25 mm internal diameter 0.25 *μ*m) (Restek, Bellefonte, US). (3) Carrier gas helium at a flow rate of 1.25 mL/min. A quadruple temperature of 180 °C and an ion source temperature of 250 °C maintained by the ISQ 7000 mass selective detector. The mass spectrometry ion source temperature was set at 230 °C. Electron impact mass spectra were achieved at acceleration energy of 70 eV. (4) One microlitre aliquot of extract was automatically injected in the split/splitless mode via an auto sampler. Fragment ions were analysed over 40–550 m/z mass range in the full scan. The filament delay of 5 min was used. (5) Raw data (retention time, peak area, analyte name, % quality) files were analysed and exported to the Access database, used for storage and retrieval, using GC-MS software (Thermo Scientific’s Mass Frontier). In addition, a wide range of de-convolution and analysis programmes were used, bundled with MS libraries that included a Mass Spectral Deconvolution System (AMDIS) with National Institute of Standard Technology (NIST) MS library (version 2.0, 2011) and Tag finder.

#### Bacterial isolation during urine ageing intervals

One millilitre of urine portion was drawn from each of the ageing urine samples for bacteriological analysis. This was carried out at an ageing interval of 4 days, for 24 days. Each urine sample was aseptically subjected to serial dilution. Aliquots of 0.1 mL, making a 10^-6^ dilution factor, were aseptically pipetted and each inoculated by spread plate method on the cysteine–lactose–electrolyte-deficient (CLED) medium (Oxoid, Basingstokes, UK medium) in threes. The plates were subsequently inverted and incubated for 24 hours at 37 °C. For purification, morphologically different colonies were sub-cultured on nutrient agar (NA) (HiMedia, Mumbai India) plates by streak plating. The isolated cultivable bacterial cultures were grouped into different groups based on their morphological appearance on CLED and NA. The groups were then preserved on glycerol stock at –20 °C in 20% (v v-1) for further analysis.

### Screening bacterial isolates for their ability to mediate production of phenols in urine samples of the selected mammals

To screen the bacterial isolates, collected, fresh urine under refrigeration was obtained and subjected to sterilisation using 0.22 nm Millipore filters. Aliquots of 50 mL sterilised urine were aseptically transferred into 500 mL Erlenmeyer flasks. A consortium of isolated morphologically different bacterial communities was inoculated in sterilised urine portion and incubation was carried out for 3 days at room temperature. Each of the isolated bacterium was also subjected to the sterilised urine separately. The involved bacteria were tested by inoculating aliquot portions of pooled fresh sterile urine with 10 *μ*L of 24-h-old cultures of the isolated bacterial cell suspension, 1 × 10^4^ colony forming unit (CFU)/mL, in a 0.9% NaCl solution to obtain a final concentration of 1 × 10^11^ CFU/mL. These treatments were then incubated at room temperature, under aerobic conditions.

The isolates, purity in each flask was established by inoculating a portion onto NA on each extraction day and examining these following a period of growth. In this analysis, blanks were used for control studies. The blanks were obtained through aseptic incubation of replica sterile urine for 3 days at room temperature. For phenols assays, 50 mL of each sample was obtained from test flasks after incubation and extracted with dichloromethane. The extracts were examined by GC-MS and the peak matching the major phenolic components of the selected animals’ urine was checked for in the chromatograms.

### Molecular characterisation of bacteria screened for phenols production

Genomic DNA (gDNA) was directly extracted from bacterial colonies obtained from a pure culture on a NA plate. The extraction and purification was done using a DNeasy Tissue Kit (Qiagen, Hilden German), following the manufacturer’s instructions. The DNA samples were then stored at –20 °C for further use. A polymerase chain reaction (PCR) amplification of the small subunit (SSU) 16S rRNA gene was carried out using 27-forward (5’-AGAGTTTGATCMTGGCTCAG-3’) and 1492-reverse (5’-CGGTTACCTTGTTACGACTT-3’) primers. The reaction was performed in a 50 *µ*L volume, containing 10×PCR buffer (5 *µ*L), 2 *µ*L each of primers, 2 mM dNTP mixture (2.5 *µ*L), Taq DNA polymerase (1 *µ*L), Dho (PCR water), up to 50 *µ*L, and template DNA (1 *µ*L). The PCR conditions were pre-denaturation at 94 °C for 5 min, 36 cycles of denaturation at 94 °C for 1 min, annealing at 54 °C for 1 min and extension at 72 °C for 2 min, and finally extension at 72 °C for 10 min. Genomic DNA (gDNA) and PCR products, 5 *µ*L each, were inspected for quality by gel electrophoresis. The 100-bp DNA ladder, for PCR products, and 1-Kb DNA ladder, for gDNA (Fischer scientific, United Kingdom [UK]), were loaded along with samples on their respective gels as molecular weight markers. The products were then visualised under ultra violet (UV) trans-illuminator light. The gels were then photographed using a digital photograph. Purification of the PCR products was carried out using Exonuclease-Shrimp alkaline phosphatase (Exo-SAP mix) reagent following the manufacturer’s instructions. After purification, the PCR products were then sequenced. The primers used for sequencing were 27F and 1492R for the 16S rRNA gene. Sequences were generated by Sanger (Capillary) sequencing, using the AB1 3730 DNA Sequencer (Applied Biosystems, US).

### Data analysis

Data obtained from the bacterial isolates ability to mediate phenols production were subjected to analysis of variance (ANOVA) using statistical analysis software (SAS) (2010). Means pair-wise comparison was carried out using Tukey’s HSD (Honestly Significant Difference) at 5% level. Generated sequences were assembled and edited using Finch Tv (Mishra et al. 2010). Consensus (contig) building was carried out using DNA Baser software (Zhang, Fu & Zhang 2012). For describing the isolates’ phylogenetic relationship, the 16S rRNA gene sequences were aligned using CLUSTAL X version 2 software (Larkin et al. 2007), together with related sequences obtained from the NCBI GenBank database using the BLAST search program (Altschul et al. 1997). The evolutionary history of the isolates was then presented in the form of a phylogenetic tree. The tree was inferred, based on the Jukes–Cantor model, by the maximum likelihood method (Jukes & Cantor 1969), at 1000 iterations, using MEGA 7 (Kumar, Stecher & Tamura 2016).

**Nucleotide sequence accession number**: The 16S rRNA sequences for the isolates in this study have been deposited in the NCBI GenBank under Accession Numbers MK123487-MK123505.

### Ethical considerations

Authority to conduct the research was approved by the National Commission for Science, Technology and Innovation (NACOSTI/P/17/73722/18052).

## Results

### Phenolic compounds in dichloromethane extracted urine

**FIGURE 1:**
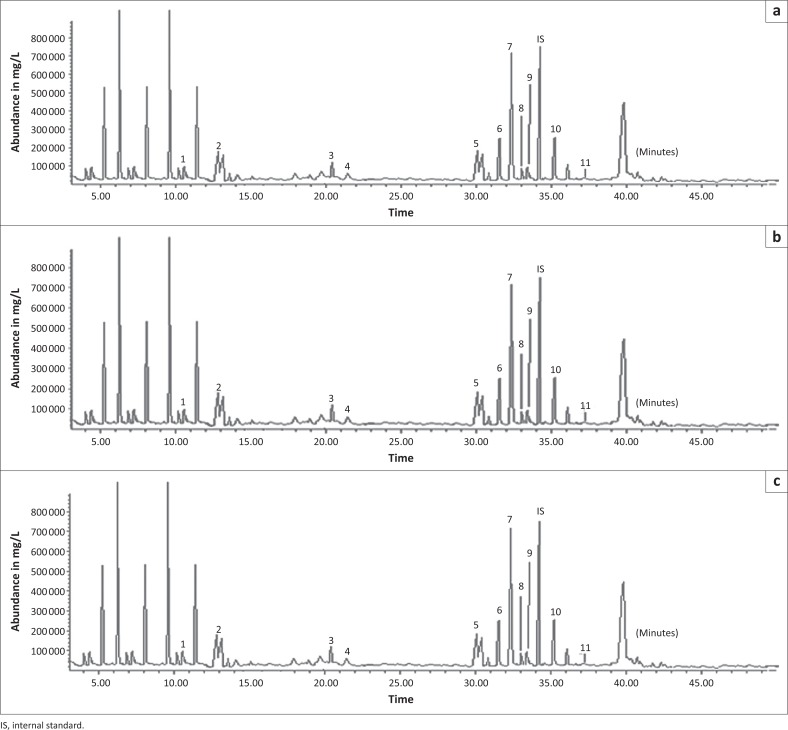
Gas chromatography-mass spectrometry chromatogram for phenolic extracts of day twenty buffalo, cattle and eland urine samples. (a) Buffalo; (b) Cattle; (c) Eland. Peaks identities: (1) 4-cresol, (2) phenol, (3) ortho-cresol, (4) 3-cresol, (5) 4-ethylphenol, (6) 3-ethylphenol, (7) 3-propylphenol, (8) 4-propylphenol and (9) 2-methyloxyphenol.

Demonstration of formation of phenols at urine ageing intervals showed several peaks in the chromatogram. The peaks indicated the presence of phenolic compounds and other non-phenolic compounds (peaks 10 and 11). Through the study, a total of nine volatile phenolic compounds were detected in the urine headspace. The compounds were identified as 4-cresol, ortho-cresol, 3-cresol, phenol, 3-ethylphenol, 3-propylphenol, 2-methyloxyphenol, 4-ethylphenol and 4-propylphenol. A representative chromatogram of the phenols identified with their peak identities is shown in Figure 1.

### Bacterial isolates ability to mediate production of phenols in mammalian urine samples

A total of 19 morphologically different bacteria colonising ageing mammalian urine were isolated. Urine fermentation with mixed bacteria (m.b) revealed the presence of eight phenolic compounds (Table 1). A comparison between individual isolates incubated in sterile urine samples and that of mixed culture samples showed some similarities in the type of phenolic compounds detected. The resultant phenolic concentrations varied, at *p* ≤ 0.05 when compared across test bacteria. Of the 19 bacteria previously isolated, eight revealed a significant association with volatile phenols (Table 1). These included b2, b22, E11, E49, B25, B34, B42 and B70. The volatile phenols of biological origin detected in urine samples incubated with these bacteria were also detected in naturally colonised mammalian urine samples (Figure 1).

**TABLE 1:** Quantitative and qualitative analysis of phenolic compounds using cattle, buffalo and elands’ sterile fresh urine incubated with various bacterial isolates.

Bacterial isolate	Phenolsʼ forms identified, mg/L (mean ± SE)							
	4-Cresol	o-Cresol	3-Cresol	3-Ethylphenol	4-Ethylphenol	3-Propylphenol	4-Propylphenol	Phenol
m.b	159542.33 ± 1735.23^d^	141549.33 ± 1735.51^a^	165767.33 ± 1767.30^a^	163582.00 ± 1766.23^b^	87753.67 ± 879.40^b^	273865.33 ± 2029.37^b^	98887.67 ± 304.80^a^	42106.67 ± 884.75^c^
b2	19.00 ± 3.51^f^	98100.67 ± 1527.20^b^	20.33 ± 2.28^c^	ND	ND	ND	ND	21.67 ± 7.22^e^
b22	28.67 ± 5.46^f^	ND	28.00 ± 2.89^c^	273865.33 ± 2029.37^a^	146816.33 ± 23331.60^a^	12.67 ± 3.48^e^	14.00 ± 4.51^c^	17.33 ± 3.33^e^
E11	425989.00 ± 3293.56^a^	26.33 ± 3.84^c^	ND	16.33 ± 4.10^c^	20.00 ± 3.0^c^	543339.67 ± 2844.94^a^	13797.33 ± 574.75^b^	133811.33 ± 3481.30^a^
E49	170206.67 ± 2848.00^c^	42.67 ± 4.45^c^	32.33 ± 3.31^c^	49.00 ± 12.12^c^	42.00 ± 11.^14c^	27.33 ± 4.06^e^	218.33 ± 63.44^c^	14589.00 ± 883.89^d^
B25	333066.00 ± 2335.57^b^	33.67 ± 1.86^c^	ND	49.33 ± 3.48^c^	44.33 ± 2.60^c^	97421.33 ± 1523.49^c^	35.33 ± 3.89^c^	122922.33 ± 580.56^b^
B34	104538.67 ± 2514.82^e^	ND	35703.33 ± 48.42^b^	ND	ND	38.67 ± 1.86^e^	ND	ND
B42	24.00 ± 1.53^f^	24.00 ± 0.58^c^	14.33 ± 0.33^c^	ND	15.33 ± 2.91^c^	27813.33 ± 11766.87^d^	25.33 ± 1.76^c^	16.67 ± 2.82^e^
B70	432655.67 ± 178.79^a^	16.67 ± 2.19^c^	ND	21.33 ± 1.41^c^	22.00 ± 2.89^c^	546006.33 ± 1731.19^a^	12797.33 ± 1152.10^b^	127144.67 ± 1455.83^b^
Blank	17.67 ± 0.88^f^	17.33 ± 1.2^c^	17.33 ± 1.20^c^	15.67 ± 1.33^c^	13.00 ± 0.58^c^	14.33 ± 1.20^e^	16.67 ± 1.45^c^	13.33 ± 0.88^e^
*p*	< 0.0001	< 0.0001	< 0.0001	< 0.0001	< 0.0001	< 0.0001	< 0.0001	< 0.0001

SE, standard error; ND, not detected; m.b, mixed bacteria for the 19 isolates; ND, (not detected) represents values that fell below the detection limit.

Note: Each value is expressed as a mean (mg/L) of three replication ±standard error; (*n* = 3).

All compounds listed above had match percent (percentage quality) > 80%.

Blank, sterile fresh urine incubated at 37 °C for 3 days.

a,b,c,d,e,f , Means followed by the same letter within a column are not significantly different according to Tukey’s Honest Significance Difference (HSD) at 5% level.

Isolates b5, b7, b10, b12, E8, E48, E51, E53, B71, B73 and B74 (not shown in the table) did not register any detectable amounts of phenols when incubated in sterilised fresh urine of the study mammals.

As shown in Table 1, para-cresol was significantly present in urine samples incubated with E11 (425989.00 mg/L), E49 (170206.67 mg/L), B25 (333066.00 mg/L), B34 (104538.67 mg/L), B70 (432655.67 mg/L) and m.b (159542.33 mg/L), at (*p* ≤ 0.0001). O-cresol was only observed in urine samples incubated with b2 (98100.67 mg/L) and m.b (141549.33 mg/L), at *p* ≤ 0.0001. The m-cresol was revealed in the presence of B34 (35703.33 mg/L) and m.b (165767.33 mg/L), at *p* ≤ 0.0001. The 3-ethylphenol was recorded when urine was subjected to b22 (273865.33 mg/L) and m.b (163582.00 mg/L) at *p* ≤ 0.0001. 4-ethylphenol was revealed in urine incubated with b22 (216816.33 mg/L) and m.b (87753.67 mg/L), at *p* ≤ 0.0001. 3-propyphenol was significantly associated with E11 (543339.67 mg/L), B25 (97421.33 mg/L), B42 (27813.33 mg/L), B70 (546006.33 mg/L) and m.b (273865.33 mg/L), at *p* ≤ 0.0001. 4-propylphenol was significant in m.b (98887.67 mg/L), E11 (13797.3 mg/L) and B70 (12797.33 mg/L), at *p* ≤ 0.0001. Phenol was significant at E11 (133811.3 mg/L), B70 (127144.67 mg/L), m.b (42106.67 mg/L), B25 (122922.33 mg/L) and E49 (14589.0 mg/L), at *p* ≤ 0.0001.

### Molecular characterisation of bacterial isolates screened for potential to mediate production of phenols

The molecular characterisation with 16S rRNA gene confirmed the bacteria to belong to different species and strains. Molecular analysis of the 16S rRNA gene of bacteria associated with phenols production authenticated the existence of a varied population of bacteria in cattle, buffalo and eland urine at different ageing intervals. Genomic DNA extracted from pure bacterial cultures showed an intact band when loaded to an 0.8% agar rose gel run at 80 V for about 30 min. The 1500 bp amplified region of 16S rRNA gene revealed different band intensities when visualised in 1.2% agar rose gel run at 80 V for 1 h. At approximately 1500 bp, all PCR products showed a definite and appropriately sized band in all lanes. On sequencing the 19 bacterial isolates, 14 different species belonging to different strains were revealed. The query sequence in the NCBI GeneBank database, using BLAST based on 16S rRNA gene sequencing, showed the relatedness of the tested organism with the same identity within different genera (Table 2).

**TABLE 2:** Bacterial strains showing significant similarity with mammalian urine bacterial isolates tested for their ability to mediate production of phenols.

Laboratory designation	Spp/strain identification†	Accession No. of the nearest neighbour	16S rRNA gene similarity (%)	Associated phenolic compound‡
b2	*Planococcus massiliensis ES2*	NR 144714.1	99	o-cresol
b5	*Providencia rettgeri* RB151	CP017671.1	99	ND
b7	*Bacillus cereus* ISSFR-3F	CP018931.1	99	ND
b10	*Bacillus pumilus* GLB197	CP018574.1	99	ND
b12	*Bacillus cereus* MLY1	CP024655.1	99	ND
b22	*Psychrobacter alimentarius* PAMC 27889	NZCP014945.1	99	3-ethylphenol/4-ethylphenol
E8	*Bacillus cereus* ATCC 4342	CP009628.1	99	ND
E11	*Enterococcus faecalis* KUB3006	AP018538.1	90	4-cresol/3-propyphenol/4-propylphenol/phenol
E48	*Bacillus megaterium* YC4-R4	CP026736.1	99	ND
E49	*Streptococcus agalactiae* 2603V/R	NC004116.1	100	4-cresol/phenol
E51	*Bacillus cereus* M3	CP016316.1	99	ND
E53	*Bacillus safensis* U17-1	CP015611.1	99	ND
B25	*Morganella morganii* subsp. *morganii* KT	NC020418.1	99	4-cresol/3-propyphenol/phenol
B34	*Micrococcus luteus* NCTC 2665	NC012803.1	99	4-cresol/3-cresol
B42	*Ochrobactrum pituitosum* AA2	CP018782.1	92	3-propyphenol
B70	*Enterococcus faecalis* OG1RF	CP002621.1	99	4-cresol/3-propyphenol/4-propylphenol/phenol
B71	*Bacillus cereus* CMCC P0021	CP011151.1	99	ND
B73	*Bacillus amyloliquefaciens* subsp. *plantarum* UCMB5033	HG328253.1	99	ND
B74	*Alcaligenes faecalis* JQ135	CP021641.1	99	ND

Note: ND (not detected) is indicated against bacterial isolates that were negative for mediation of phenols when inoculated in sterilised fresh urine of the study animals.

† , Best match in NCBI GeneBank database.

‡ , Phenols detected in fresh urine samples inoculated with specific bacteria for 3 days at ambient conditions.

As shown in Table 2, the phylogenetic relationship of the isolates revealed the presence of the *Planococcus massiliensis strain ES2* (99% 16S rRNA gene sequence similarity with isolate b2); *Psychrobacter alimentarius* PAMC 27889 (99% 16S rRNA gene sequence similarity with isolate b22); *Providencia rettgeri* RB151 (99% 16S rRNA gene sequence similarity with isolate b5); *Enterococcus faecalis* OG1RF (99% 16S rRNA gene sequence similarity with isolate B70); *Enterococcus faecalis* KUB3006 (90% 16S rRNA gene sequence similarity with isolate E11); *Morganella morganii* subsp. *morganii* KT (99% 16S rRNA gene sequence similarity with isolate B25); *Micrococcus luteus* NCTC 2665 (99% 16S rRNA gene sequence similarity with isolate B34); *Ochrobactrum pituitosum* AA2 (92% 16S rRNA gene sequence similarity with B42); *Alcaligenes faecalis* JQ135 (99% 16S rRNA gene sequence similarity with isolate B74), *Streptococcus agalactiae* 2603V/R (99.9% 16S rRNA gene sequence similarity with isolate E49), *Bacillus pumilus* GLB197 (99% 16S rRNA gene sequence similarity with isolate b10); *Bacillus cereus* MLY1 (99% 16S rRNA gene sequence similarity with isolate b12); *Bacillus cereus* CMCC P0021 (99% 16S rRNA gene sequence similarity with isolate B71); *Bacillus amyloliquefaciens* subsp. *plantarum* UCMB5033 (99% 16S rRNA gene sequence similarity with isolate B73); *Bacillus cereus* ATCC 4342 (99% 16S rRNA gene sequence similarity with isolate E8); *Bacillus cereus* ISSFR-3F (99% 16S rRNA gene sequence similarity with isolate b7); *Bacillus cereus* M3 (99% 16S rRNA gene sequence similarity with isolate E51); *Bacillus megaterium* YC4-R4 (99%16S rRNA gene sequence similarity with isolate E48) and *Bacillus safensis* U17-1 (99% 16S rRNA gene sequence similarity with isolate E53).

**FIGURE 2:**
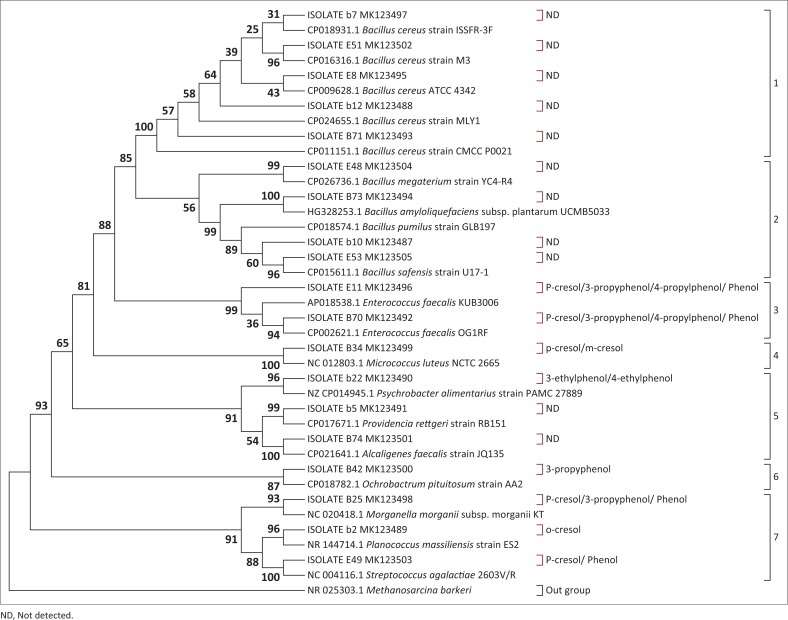
Molecular phylogenetic analysis of bacterial communities with and without the potential to mediate production of phenols in mammalian urine by the maximum likelihood method.

Evolutionary relatedness of the 19 bacterial isolates screened for potential to mediate production of phenols in mammalian urine was evaluated based on their 16S rRNA gene. A phylogenetic tree based on these isolates is shown in Figure 2. The bacteria were grouped into seven clusters. The clusters were grouped based on isolates similarity in relation to mediation of urine volatile phenols. Furthermore, some bacterial communities were clustered depending on when they were isolated from urine, whereas others were grouped along their genotypic relatedness.

Clusters 1 and 2 exclusively contain bacterial communities that showed negative results for phenol mediation. Also, worthy of note is that bacteria that fell under these clusters were isolated between days, 16 and 24 of ageing mammalian urine. The two clusters were supported by bootstrap values of 100% and 56%, respectively. Cluster 1 comprised of isolates b7, E51, E8, b12 and B71, whereas cluster 2 was represented by isolates E48, B73, b10 and E53. The closed neighbours for all the isolates in these two clusters were dominated by different members of the *Bacillus* spp.

Bacteria having ability to mediate formation of phenols in mammalian urine were grouped in closely related phylogenetic positions. They were grouped together in clusters 3, 4, 6 and 7. The clusters were supported by bootstrap values of 99%, 100%, 87% and 91%, respectively. Cluster 3 was represented by isolates E11 and B70. These isolates were closely related to different strains of *Enterococcus faecalis*. Clusters 4 and 6 were represented by only one isolate each, B34 and B42, respectively. The close neighbour for isolate B34 was *Micrococcus luteus* NCTC 2665, while that for B42 was *Ochrobactrum pituitosum* AA2. Cluster 7 comprised of three isolates, B25, b2 and E49, whose close neighbours were *Morganella morganii* subsp. *morganii* KT, *Planococcus massiliensis strain ES2* and *Streptococcus agalactiae* 2603V/R in that order.

Cluster 5, supported by a bootstrap value of 91%, comprised of isolates b22, b5 and B74 represented by closest neighbours, *Psychrobacter alimentarius* PAMC 27889, P*rovidencia rettgeri* RB151 and *Alcaligenes faecalis* JQ135, respectively. Nevertheless, a unique trend was noted with this cluster. Not all the representative isolates were positive for mediation of phenols production. Isolates b5 and B74 showed negative results when tested for potential to mediate production of phenols in mammalian urine (Table 1). In terms of genotypic relatedness, these isolates were distantly related to their counterparts in clusters 1 and 2.

The evolutionary history was inferred by using the maximum likelihood method based on the Jukes–Cantor model (Jukes & Cantor 1969). The bootstrap consensus tree inferred from 1000 replicates (Felsenstein 1985) is taken to represent the evolutionary history of the taxa analysed (Felsenstein 1985). The percentage of replicate trees in which the associated taxa clustered together in the bootstrap test (1000 replicates) is shown next to the branches (Felsenstein 1985). Initial tree(s) for the heuristic search were obtained automatically by applying Neighbour-Join and BioNJ algorithms to a matrix of pair-wise distances estimated using the maximum composite likelihood approach and then selecting the topology with a superior log likelihood value. The analysis involved 39 nucleotide sequences. There were a total of 1494 positions in the final dataset. Evolutionary analyses were conducted in MEGA7 (Kumar et al. 2016). One representative sequence from each urine isolate Operational Taxonomic Units (OTUs) was included in the tree along with the sequence from the NCBI database. ND (not detected) is indicated against isolates that were negative for phenols production. Bacterial isolates with potential to mediate phenols production are indicated against their associated phenols in brackets. The far left values, from 1 to 7, show the number of clusters.

## Discussion

### Phenolic compounds in dichloromethane extracted urine

The presence of phenolic compounds in DCM urine extracts of this study confirmed that mammalian urine has a large range of phenolic compounds. These findings are in agreement with previous studies that identified phenols in animal urine (Alkhaldy et al. 2018; Spiehs et al. 2018; Tangtrakulwanich et al. 2015). The presence of tsetse attractant cues in eland ageing urine provides more information on which animals to target when controlling tsetse. Eland have been shown to be resistant to trypanosomiasis (Pappas 2002). However, it has not been known if the animal’s urine has the potential to attract tsetse. Some of the phenolic compounds detected in this study, for instance, phenol, 4-cresol and 3-propylphenols, have been previously linked to morsitans flies behavioural response (Brightwell, Dransfield & Kyorku 1991; Hassanali et al. 1986; Madubunyi et al. 1996; Owaga et al. 1988; Vale, Hall & Gough 1988).

### Bacterial isolation at urine ageing intervals

This study was able to cultivate 19 morphologically different bacteria in ageing urine samples when compared to the study carried out by Íñigo et al. (2016). In their research, they sought to characterise microbes found in human urine using culture independent methods. They reported 336 bacterial species in the urine samples. The lower number of bacteria identified in this study, as compared to Íñigo et al.’s (2016) findings, can be attributed to the variation in the species under study or in the methods used to culture and identify the isolates.

### Bacterial isolateʼs ability to mediate production of phenols in urine samples of the selected mammals

When the sterilised fresh urine was fermented with a mixture of isolated bacteria, the amounts of detected phenols were varied. In a study involving mixed bacterial cultures and microbes in soil, Kai et al. (2009) observed that the cultures had potential to produce diverse volatile organic compounds. The differences in the concentration of phenols, in this study, can be attributed to multivariate factors. For example, there must have been competition among the mixed bacteria, where some inhibited activities of their neighbours who might have been responsible for mediation of phenols production. Andreev et al. (2017) documented a study related to this suggestion. Some of the bacteria could also not be producing any volatiles but are essential for the existence of others that mediate phenols production in urine. Variation in the amounts of detected phenols can also be attributed to the presence of microbes that naturally prevent the formation of phenols. This is likely achieved through enzymatic activities of the associated bacteria. The activities work against the production of phenols by other species. Such a phenomenon has been reported by Troccaz et al. (2013). Urine inoculated with a consortium of bacteria may be comprising bacteria that mediate production of detected phenols as well as those that break the phenols down once they are generated. These findings suggest that different bacterial communities co-existing in urine are essential determinants for the release of volatile organic compounds’ complexes needed to elicit tsetse’s behavioural response.

When the sterilised urine samples were inoculated with individual bacteria for 3 days, the results revealed a potential of eight isolates to mediate production of volatile phenols. These findings concur with previous studies (Schulz & Dickschat 2007; Troccaz et al. 2013). Troccaz et al. (2013) profiled different bacterial volatiles in human urine while in their study that sought to establish different volatiles emitted by bacteria, Schulz and Dickschat (2007) associated about 65 percent of bacteria investigated with production of the volatiles. In addition, Tasin et al. (2018) associated volatiles that determine the behavioural role of insects to microorganisms. The presence of volatile phenols from specific bacteria inoculated in urine samples may be attributed to multiple factors. Firstly, it might be because of a difference in nutritional activities of different bacterial species inoculated in urine samples as suggested by Tasin, Knudsen and Pertot (2012) and Teira et al. (2011). In a nutrients manipulation experiment, Teira et al. (2011) demonstrated that bacterial phylogenetic groups react differently to an alteration in nutrients. Secondly, the bacterial individual metabolism could be linked to a difference in the phenol’s profiles observed. By the use of metabolic pathways of glycolysis, or degradation of amino acids, bacteria are able to generate aromatic compounds (Schulz & Dickschat 2007). According to Todar (2012), many volatile organic compounds are generated during primary and secondary metabolism in microorganisms. Therefore, the production of secondary metabolic phenols in mammalian urine can be said to be limited to a certain phylogenetic group or are species-specific.

### Characterisation of bacterial isolates with potential to mediate production of tsetse attractive phenols in mammalian urine

The significant similarity of bacterial isolates that was positive for mediation of production of phenols, with *Enterococcus faecalis* KUB3006, *Psychrobacter alimentarius* PAMC 27887, *Streptococcus agalactiae* 2603V, *Morganella morganii* sub.sp. *morganii* KT, *Micrococcus luteus* NCTC2665, *Planococcus massiliensis ES2, Ochrobactrum pituitosum* AA2 and *Enterococcus faecalis* OGIRF suggests the potential of these bacterial communities to mediate production of phenols in mammalian urine. These findings are supported by earlier studies by Alkhaldy et al. (2018), Schoefer et al. (2003), Schneider and Blaut (2000), Schneider et al. (1999) and Bokkenheuser, Shackleton and Winter (1987) who demonstrated the ability of different bacteria to metabolise phenols and other volatiles.

In this study, bacteria having the ability to mediate the formation of phenols were clustered in closely related phylogenetic positions. This clustering suggests that bacterial communities with potential to mediate production of phenols are restricted to a limited phylogenetic group. Therefore, the bacteria may have evolved to colonise mammalian urine where they mediate production of volatile phenols that influence the tsetse’s host-seeking behaviour. In addition, biochemical pathways that are responsible for the production of phenols by bacterial species may be conserved across taxonomic groups of these bacteria. Isolate b2, which was closely related to *Psychrobacter alimentarius* PAMC 27887, may have retained critical genes that code for the mediation of production of phenols. Unlike its counterparts in that cluster, this isolate showed potential for the mediation of production of phenols.

*Enterococcus faecalis* strains showed potential to produce 4-cresol, phenol and 3-propylphenol. These phenols have been previously reported to attract tsetse to their host animals (Bursell et al. 1988; Gikonyo et al. 2000; Hassanali et al. 1986). These results closely relate to a study carried out by Trocazz et al. (2013) who documented the role of *Enterobacteriaceae* in the production of p-cresol, phenol and other volatiles in human urine. The origin of *Enterococcus faecalis* varies from animals, environmental and human sources. However, the intestinal tract of animals and humans is the natural habitat of this bacterium (Klein 2003). The bacterium is tolerant to a wide range of stress and can survive for a long time outside their natural intestinal hosts (Arias & Murray 2012). This long interaction with fermenting urine provides a platform for the generation of urine phenols. Production of phenols suggests this bacterium’s ecological importance in the tsetse’s host-seeking behaviour and transmission of AAT.

*Morganella morganii* sub.sp. *morganii*, just like *Enterococcus faecalis* strains, showed the potential to generate 4-cresol, phenol and 3-propylphenol. These phenols have an ecological role in the attraction of tsetse vectors to the host (Bursell et al. 1988). These findings are comparable to a study carried out by Marshall et al. (2016), Engl and Kaltenpoth (2018). In their study, the researchers established that *Morganella morganii* produces phenol as a sex pheromone of the New Zealand grass grub (*Costelytra zealandica*). *Morganella morganii* is often found in the human gut and animal gut (Jones-Dias et al. 2016). The bacterium has also been found commonly in the livestock environment (Fischer et al. 2016; Rodrigues et al. 2017; Wang et al. 2017). Through its interaction with animals, it is likely that this bacterium gains access to mammalian urine where it mediates production of associated urine phenols.

In this study, *Streptoccoccus agalactiae* 2603V bacterium showed the potential to mediate production of phenol and 3-propylphenol which are known to attract tsetse. The incidences of this bacterium in the study samples compares well with other findings. For example, production of 3-methylbutanoic acid volatiles in sterilised milk strongly correlated with the growth of S*taphylococcus aureus* when mixed with S*taphylococcus agalactiae* (Chen et al. 2018). The bacterium has been isolated in different bovine environments (Holmøy et al. 2018; Miranda et al. 2018; Reyes et al. 2017; Skjstrup et al. 2018; Svennesen et al. 2019). There is a possibility that the prevalence of this bacterium in the bovine environment creates an opportunity for the bacterium to colonise mammalian urine and thus mediate phenols’ formation.

*Micrococcus leteus,* NCTC 2665, in this study, showed the ability to generate 4-cresol and 3-cresol. Previous studies, for example, field experiments involving tsetse behavioural responses by Hassanali et al. (1986) profiled 4-cresol from the excretory products of buffalo as a fundamental component of the tsetse host location. Mediation of the production of phenols, especially 4-cresol, by this bacterium in this study may explain earlier reports of the involvement of skin bacteria in the production of volatiles that determine the host-seeking behaviour of some insects (Verhulst et al. 2011). The *Micrococcus* spp. main natural habitat is mammalian skin; the secondary habitat is dairy products, meat, soil and water (Grice et al. 2009; Grice & Segre 2011). In related studies that targeted the analysis of mosquito attractive cues in animals and human skin bacteria, Busula et al. (2017) reported the ability of volatiles, generated by bacteria on cow skin, to attract mosquito species. It is therefore noteworthy to suggest that this bacterium plays a role in the production of phenols in wildlife and livestock when urine comes into contact with animal skin.

This study established that *Psychrobacter alimentarius* PAMC 27889 constitutes a proportion of ageing mammalian urine flora. The bacterium showed the ability to generate 3- and 4-ethylphenols. The 3-ethylphenol has been previously attributed to the attractiveness of cattle urine to *G. pallidipes* Austen and *Glossinna morsitans morsitans* Westwood (Bursell et al. 1988). A study carried out by Broekaert et al. (2013) established sea food spoilage to volatile compounds associated with *Psychrobacter* spp. The researchers established volatile compounds that were linked to *Psychrobacter* spp. In a related study, Trexler et al. (2003) reported that gravid *Aedes abopictus* oviposited more often in water inoculated with a member of *Psychrobacter* spp. Members of *Psychrobacter* spp. have been previously isolated in diverse environments (Hamm et al. 2016; Meziti et al. 2010; Yang et al. 2017). The association of *Pyschrobacter* spp. with bovine environment could be a favourable prospect for *Psychrobacter alimentarius* PAMC 27889 to colonise mammalian urine, which supports the formation of urine phenols.

*Ochrobactrum pituitosum* AA2 bacteria were found to have the potential of generating 3-propylphenol, which is a critical compound of tsetse attraction to the host. A study involving rhizospheres profiled this bacterium as having the ability to produce volatile organic compounds that promote plant growth (Baysal & Silme 2017). Members of *Ochrobactrum* spp. have been isolated from several ecological niches, for instance, water, soils, animals, plants and humans (Dini-Andreote et al. 2014; Kulkarni et al. 2017).

*Planococcus massiliensis* strain *ES2,* in this study, was found to have the potential to generate o-cresol when inoculated in sterilised urine. Currently, it is not clear if the o-cresol has the potential to attract tsetse. Brega et al. (1990) documented the presence of this particular phenolic compound in human urine even though the presence was not linked to this bacterium. This bacterium was previously isolated in the human gut, sea water, clamp prawns and the marine environment (Seck et al. 2016; Too et al. 2017). The ability to survive in different environments may be related to their presence in the fermenting urine of mammals where they are associated with the production of o-cresol as established in this study.

## Conclusion

This study sought to profile bacterial communities in mammalian urine that mediate the production of tsetse attractive phenols. The study revealed that some bacterial communities colonising mammalian urine seem to be well characterised by certain volatile phenolic compounds. The bacteria identified, that appeared to mediate production of tsetse attractive phenolic compounds in mammalian urine, include *Psychrobacter alimentarius* PAMC 27887, *Enterococcus faecalis* KUB3006, *Streptococcus agalactiae* 2603V, *Morganella morganii* sub.sp. *morganii* KT, *Micrococcus luteus* NCTC 2665, *Ochrobactrum pituitosum* AA2 and *Enterococcus faecalis* OGIRF. The study opens potential pathways of reducing the attraction of savannah tsetse to host animals and enhancing ‘push–pull’ models in vector control that combines the use of repellents on host animals and attraction to traps. This study hypothesises that tsetse attraction to livestock and wildlife can be controlled significantly by achieving an optimum solution in the reduction of bacteria that mediate the production of phenols. This may enhance the controlled release of the semiochemicals in the grazing fields. However, more research needs to be carried out to substantially reduce the attraction of tsetse to their host animals.
